# Occlusion of the Vagina Relieved by an Operation

**Published:** 1851-09

**Authors:** 


### SELECTIONS

FROM

### FOREIGN AND HOME MEDICAL JOURNALS.

---

*Occlusion of the Vagina relieved by an operation.*—In the *American Jour. Med. Sci.*, for July, Dr. J. Mason Warren reports three cases of occlusion of the vagina, one congenital, the other two the result of inflammation after child-bearing, in which he resorted successfully to the use of the knife. One of the operations is thus described:

The patient being fully etherized with chloric ether, an incision was made transversely across the mucous membrane of the lower part of the vagina. This disclosed muscular fibres, which being carefully divided through the aperture thus made, a delicate membrane of dark color protruded. It was suggested by one of the gentlemen present that this might possibly be the peritoneum, which in a case of malformation and non-existence of the vagina might take an abnormal direction. For the purpose of testing this, I attempted to separate it from the surrounding textures, knowing the loose character of the cellular tissue which attaches the peritoneum to the neighboring organs and the pelvis. This was at once found to be impracticable, and on renewal of the effort the resisting part yielded, and the finger passed through into what appeared at first to be the abdominal cavity, so well defined was the anatomy of the walls of the pelvis. The absence of intestines, and the appearance of a small quantity of dark-colored fluid by the side of the finger, soon made it evident that the vagina had been opened.

The size of the cavity occupying the entire pelvis, and the complete absence of os uteri or other boundary, between the uterus and vagina, was on examination sufficiently evident to all present.

By the aid of slight pressure on the abdomen, about half a pint of thick, tenacious fluid escaped. As the uterus did not at once take on contractions, no further efforts were made to evacuate the fluid, but a bit of sponge was introduced into the opening to prevent the parietes from adhering. The vegetations at the orifice of the urethra were now removed by the scissors, and the base of the tumors cauterized with nitrate of silver. To show the extreme sensibility of these tumors, it may be observed that as soon as they were interfered with the patient, although well etherized and perfectly passive through all the previous operation, immediately drew back as if in extreme pain.

At 7 P. M., she was in good spirits, and expressed herself entirely relieved by the operation. The effects of the ether had passed off, notwithstanding she had been kept for three-quarters of an hour fully under its influence. I warned her as to the great danger she incurred from any irregularity in diet or exposure to cold, as I found her disposed to leave her bed, and she was demanding food.

On the 14th September, the day following the operatian, she was reported to have passed a good night. The sponge was removed from the vagina, and a free discharge of the peculiar fluid took place; after a few hours it was again introduced. No urine had been passed since the operation; during the succeeding night, however, a copious evacuation of the bladder took place.

On the 17th, she still continued to improve, and the tumor of the abdomen to diminish. The finger passed into the vagina could distinguish the os uteri, as it were, gradually forming itself. It was about the size of a tumbler, with thick edges, and covered with dilated blood-vessels. The sponge tent when withdrawn was very offensive.

As she was urgent to go among her friends, I agreed to-day, the 20th, that she should do so; being conveyed to the rail-road in a carriage with care, and kept in a recumbent position until she arrived at the point of her destination. She was then to remain a few weeks longer in bed, or on a sofa, without attempting to use any exercise. At the period of leaving town, she was quite well. The urine was passed naturally and without pain, the sensitive tumors of the urethra having been destroyed by the operation. The discharge from the vagina had partially ceased or had been replaced by a serous exudation. Her appetite and the state of her digestive organs were natural.

